# Identification and Immunocytochemical Characterization of Piccolino, a Novel Piccolo Splice Variant Selectively Expressed at Sensory Ribbon Synapses of the Eye and Ear

**DOI:** 10.1371/journal.pone.0070373

**Published:** 2013-08-06

**Authors:** Hanna Regus-Leidig, Corinna Ott, Martina Löhner, Jenny Atorf, Michaela Fuchs, Tina Sedmak, Jan Kremers, Anna Fejtová, Eckart D. Gundelfinger, Johann H. Brandstätter

**Affiliations:** 1 Department of Biology, Animal Physiology, Friedrich-Alexander-University Erlangen-Nuremberg, Erlangen, Germany; 2 Department of Ophthalmology, University Hospital Erlangen, Erlangen, Germany; 3 Leibniz Institute for Neurobiology, Magdeburg, Germany; 4 Center for Behavioral Brain Science, Magdeburg, Germany; University of Oldenburg, Germany

## Abstract

Piccolo is one of the largest cytomatrix proteins present at active zones of chemical synapses, where it is suggested to play a role in recruiting and integrating molecules relevant for both synaptic vesicle exo- and endocytosis. Here we examined the retina of a Piccolo-mutant mouse with a targeted deletion of exon 14 in the *Pclo* gene. Piccolo deficiency resulted in its profound loss at conventional chemical amacrine cell synapses but retinal ribbon synapses were structurally and functionally unaffected. This led to the identification of a shorter, ribbon-specific Piccolo variant, Piccolino, present in retinal photoreceptor cells, bipolar cells, as well as in inner hair cells of the inner ear. By RT-PCR analysis and the generation of a Piccolino-specific antibody we show that non-splicing of intron 5/6 leads to premature translation termination and generation of the C-terminally truncated protein specifically expressed at active zones of ribbon synapse containing cell types. With *in situ* proximity ligation assays we provide evidence that this truncation leads to the absence of interaction sites for Bassoon, Munc13, and presumably also ELKS/CAST, RIM2, and the L-type Ca^2^^+^ channel which exist in the full-length Piccolo at active zones of conventional chemical synapses. The putative lack of interactions with proteins of the active zone suggests a function of Piccolino at ribbon synapses of sensory neurons different from Piccolo’s function at conventional chemical synapses.

## Introduction

Neurotransmission at chemical synapses is restricted to specialized areas of the presynaptic plasma membrane called active zones (AZ). There, a tight network of multi-domain scaffolding proteins, the cytomatrix at the AZ (CAZ), orchestrates the controlled exo- and endocytosis of synaptic vesicles in space and time. CAZ components like Bassoon (Bsn), Piccolo/Aczonin (Pclo), RIM, ELKS/CAST, and Munc13 contribute to synaptic transmission either by directly participating in vesicle priming, docking, and retrieval, or by providing interaction sites for molecules involved in these processes [1], [2]. Morphological variations of the AZ are the ribbon synapses of sensory neurons of the visual and auditory systems [3]. Whereas the CAZ at conventional chemical synapses is a more or less two-dimensional specialization, ribbon synapses harbor a three-dimensional CAZ, the synaptic ribbon, for the continuous and graded release of neurotransmitter.

The photoreceptor synaptic ribbon is an electron-dense plate-like structure, anchored to the presynaptic plasma membrane and extending several hundred nm into the cytoplasm. It tethers hundreds of synaptic vesicles and transmits changes in light intensity via graded modulation of glutamate release [4], [5]. Although the importance of the synaptic ribbon in neurotransmission has been proven, its exact functional contribution is not well understood [6]–[8]. One approach to decipher the ribbon’s role in neurotransmission is to identify and characterize its molecular components. The photoreceptor ribbon complex can be subdivided into two spatially and functionally separate compartments, the arciform density and the ribbon with its associated proteins [9]. The arciform density consists of a dense protein network adjacent to the presynaptic plasma membrane, which comprises RIM2 [9], [10], ELKS/CAST [9]–[12], and ubMunc13-2 [13]. The major constituent of the ribbon is the ribbon-specific protein RIBEYE [14]; CtBP1, RIM1, KIF3A, and Pclo are reported to be associated with the ribbon [9], [10], [15], [16].

In the last years, increasing efforts have been made to elucidate the role of the cytomatrix protein Pclo at AZs of chemical synapses, but its relevance in neurotransmission is still far from being clear. The tight spatial overlap of the two paralogous proteins Pclo and Bsn at conventional chemical synapses [10] and their ability to directly interact with each other and with partially the same AZ components [1], [17] implies a high degree of redundancy between the two proteins. At ribbon synapses, Pclo and Bsn are spatially segregated and thus might have adopted different tasks in synaptic transmission [9], [10], [16]. In line with this, Bsn-deficient photoreceptors show a strong synaptic phenotype with detached synaptic ribbons and impaired synaptic transmission, a phenotype which cannot be compensated by Pclo [6]. To analyze the function of Pclo at ribbon synapses, we studied the retina of a Pclo-mutant mouse with a targeted deletion of exon 14 of the *Pclo* gene, which causes an almost complete deficiency of full-length Pclo in the murine brain [18]. Ribbon synapses did not differ in Pclo expression between Pclo-mutant and wild-type (wt) mice, and structural and functional examination failed to uncover a ribbon synaptic phenotype. Further analysis revealed the presence of a shorter, ∼330-kDa ribbon-specific Pclo variant, which we named Piccolino. Because of a C-terminal truncation, Piccolino presumably lacks the interaction sites for RIM, Munc13, ELKS/CAST, and the L-type Ca^2^^+^ channel suggesting a function of Piccolino independent from the protein network of the arciform density/plasma membrane.

## Materials and Methods

### Ethics Statement

The experiments were performed in compliance with the guidelines for the welfare of experimental animals issued by the Federal Government of Germany and the FAU Erlangen-Nuremberg. The animal experiments were approved and registered by the Amt für Veterinärwesen der Stadt Erlangen (AZ: TS - 10/07 Lehrstuhl für Zoologie-Tierphysiologie). Mouse breeding was performed in the animal facilities of the FAU University of Erlangen-Nuremberg according to European and German (Tierschutzgesetz) guidelines for the welfare of experimental animals (AZ 820-8791.2.63). All animal experiments were performed in compliance with the guidelines issued by the University of Erlangen-Nuremberg and of the German Federal State of Sachsen-Anhalt, in accordance with the European Communities Council Regulations.

### Animals

Adult (age 2–4 months) Sprague-Dawley rats, C57BL/6 mice, PcloΔEx14 mice (*de novo* generated by crossing the B6;129S6-Pclo^tm2Sud^/J with Tg(CMV-cre)1Nagy deleter mouse strain), Tg(Rac3-EGFP)JZ58Gsat/Mmcd (Rac3-EGFP) mice, and Tg(Lrrc55-EGFP)KS290Gsat/Mmcd (Lrrc55-EGFP) mice, maintained on a 12/12 hour light/dark cycle with light on at 6 am, were used. The latter two strains were obtained from the Mutant Mouse Regional Resource Center, a NCRR-NIH funded strain repository, and were donated to the MMRRC by the NINDS funded GENSAT BAC transgenic project. B6;129S6-Pclo^tm2Sud^/J mice were purchased from Jackson Laboratory. Animals were sacrificed between 3 and 6 hours after light onset. In experiments comparing PcloΔEx14 mice with wild-type mice, wild-type animals were littermate controls from heterozygous breeding.

### Retina Preparation for Light Microscopic Immunocytochemistry

Preparation of retinal tissue and antibody incubation for light microscopic immunocytochemistry was performed as described previously [6], [9]. Briefly, the eyes were opened and retinae were immersion fixed in the eyecup for 15 or 30 min in 4% paraformaldehyde (PFA) in phosphate buffer (PB; 0.1 M, pH 7.4). Retinae were mounted in freezing medium (Reichert-Jung, Bensheim, Germany), and 12–16 µm thick vertical sections were cut with a cryostat (Leica CM3050 S, Leica, Wetzlar, Germany). Primary antibody incubation was carried out overnight at room temperature, secondary antibody incubation for 1 h. For characterization of the Pclo 49 antibody, 1 µl of the antibody was preincubated for 1 h with an excess of purified peptide. For analysis, labeled sections were examined with a Zeiss Axio Imager Z1 equipped with an ApoTome (Zeiss, Oberkochen, Germany). Images were taken with a 20x (0.8, Apochromat) or a 100x (1.3 oil, Plan-Neofluar) objective as stacks of multiple optical sections, and projections were calculated with the AxioVision 4.8 software (Zeiss). Images were adjusted for contrast and brightness using Adobe Photoshop CS (Adobe, San Jose, CA, USA).

### Cochlea Preparation for Light Microscopic Immunocytochemistry

Immunocytochemistry was performed on whole-mount preparations of the organ of Corti. The cochleae were removed from the temporal bone, carefully opened, and fixed in 4% PFA for 1 h on ice. After washing in PB, the organ of Corti was carefully removed. Primary antibody incubation was carried out overnight at 4°C, secondary antibody incubation for 1 h at room temperature. Images were taken with a Zeiss LSM 710 in combination with the Zen 2010 software (Zeiss) with a 63x (1.40 oil, Plan-Apochromat) objective as stacks of multiple optical sections, and projections were calculated with the AxioVision 4.8 software (Zeiss). Images were adjusted for contrast and brightness using Adobe Photoshop CS (Adobe).

### Antibodies

The following primary antibodies were used for retinal tissue: Monoclonal mouse anti-Bassoon mab7f (PLA 1∶2,500; Stressgen, MI, USA), mouse anti-CtBP2/RIBEYE (ICC 1∶10,000; BD Biosciences, Heidelberg, Germany), mouse anti-panMunc13 (PLA 1∶100; BD Biosciences), polyclonal rabbit anti-Pclo 4 (WB 1∶1,000; [19]), rabbit anti-Pclo 6 (WB/ICC/PLA 1∶500–1∶1,000; generated against a purified protein corresponding to aa 4444–4586 of rat Pclo), rabbit anti-RIBEYE (ICC/PLA 1∶500–1∶1,000; Synaptic Systems, Göttingen, Germany), guinea pig anti-Pclo 44a (WB 1∶1,000; ICC 1∶4,000; [16]). Polyclonal rabbit antibody against Piccolino (Pclo 49; WB 1∶5,000; ICC/PLA 1∶5,000–1∶10,000) was generated by BioTrend (Cologne, Germany). Single peptides representing the first 23 amino acids of intron 5/6 in the *Pclo* gene (GQYDVAIDPALNCHYGVMHLVSG) were used for immunization over 35 days. From the final bleeding, the serum was affinity purified against the peptide, and the purified antibody was dialyzed against PBS.

For whole-mount preparations of the organ of Corti the following antibody concentrations were used: Monoclonal mouse anti-CtBP2/RIBEYE (1∶500), polyclonal rabbit anti-Pclo 6 (1∶100), rabbit anti-Pclo 49 (1∶500), guinea pig anti-Pclo 44a (1∶1,000). Nuclei were stained with DAPI (4,6-diamidino-2-phenylindole; 1∶50,000; Sigma-Aldrich, St. Louis, MO, USA).

The following secondary antibodies were used: Alexa™ 488 and Alexa™ 568 goat anti-mouse, goat anti-rabbit, and goat anti-guinea pig IgG conjugates (1∶500; Molecular Probes, Eugene, OR, USA), Cy5 goat anti-mouse IgG conjugate (1∶100; Dianova, Hamburg, Germany), HRP goat anti-rabbit, and goat anti-guinea pig IgG conjugate (1∶10,000; Sigma-Aldrich).

### Western Blot Analysis

For Western blots of retina and cortex synaptosomal (P2) fractions, tissues were homogenized in lysis buffer (320 mM Saccharose, 4 mM Hepes, pH 7.5) and centrifuged at 1,000×g for 10 min. The supernatants (S1) were centrifuged at 20,000×g for 20 min. Pellets (P2) were washed and dissolved in sample buffer. Equal amounts (25 µg/lane) of protein were separated by SDS-PAGE using 3–8% NuPAGE Novex Tris acetate gels (Invitrogen, Darmstadt, Germany), and transferred to PVDF membranes by tank blotting (Trans-Blot Cell, Bio-Rad Laboratories, Munich, Germany). For immunodetection, membranes were blocked with skimmed milk powder and incubated with primary antibodies overnight at 4°C. For characterization of the Pclo 49 antibody, 1 µl antibody was preincubated for 1 h with an excess of purified peptide. HRP-coupled secondary antibodies were visualized by chemiluminescent detection (Luminata™ Forte, Millipore, Schwalbach/Ts, Germany). Images were obtained with a molecular imager (ChemiDoc XRS, Bio-Rad Laboratories), and adjusted for contrast and brightness using Adobe Photoshop CS (Adobe).

### In situ Proximity Ligation Assay (PLA)

The following PLA components were purchased from Olink (Uppsala, Sweden): Duolink PLA probe anti-rabbit PLUS, Duolink PLA probe anti-mouse MINUS and Duolink in situ Detection Reagent Red. PLAs were performed according to the manufacturer. In brief, 12 µm thick cryosections were incubated overnight at room temperature with primary antibodies. Next, combinations of the PLA probes (anti-rabbit PLUS probe, anti-mouse MINUS probe, diluted in antibody dilution) were added to the sections for 1–2 h at room temperature. Ligation was performed for 30 min, followed by the amplification step for 100 min at 37°C. In order to verify correct antibody binding, the antibody mixture used for the PLA was tested in fluorescence stainings on a different set of slices.

### Electron Microscopy

For conventional electron microscopy and good tissue preservation, retinae were fixed in 4% PFA and 2.5% glutaraldehyde for 2 hours at room temperature, followed by incubation in 2% osmiumtetroxide for 1.5 hours, and retinae were embedded in Epon resin (Fluka, Buchs, Switzerland). For pre-embedding immunoelectron microscopy, retinae were prefixed in 4% PFA in Soerensen buffer (0.1 M Na_2_HPO_4_⋅2 H_2_O, 0.1 M KH_2_PO_4_, pH 7.4) for 50 minutes at room temperature and further processed as described [20], [21]. Briefly, after four cycles of freezing in liquid nitrogen and thawing at 37°C, retinae were PBS washed and embedded in buffered 2% Agar. Agar blocks were cut in 50 µm sections with a vibratome (Leica VT 1000 S, Leica). The sections were incubated in 10% normal goat serum, 1% bovine serum albumin in PBS for 2 hours, followed by incubation with primary antibodies for 4 days at 4°C. PBS washed sections were incubated with biotinylated secondary antibodies, and visualized by Vectastain ABC-Kit (both from Vector Laboratories, Burlingame, CA, USA). Sections were fixed in 2.5% glutaraldehyde in 0.1 M cacodylate buffer (pH 7.4). Diaminobenzidine precipitates were silver enhanced and postfixed in 0.5% OsO_4_ in 0.1 M cacodylate buffer at 4°C. Dehydrated specimens were flat-mounted between ACLAR®-films (Ted Pella Inc., Redding, CA, USA) in Epon resin (Fluka). For analysis, ultrathin sections were examined and photographed with a Zeiss EM10 electron microscope (Zeiss) and a Gatan SC1000 OriusTM CCD camera (GATAN, Munich, Germany) in combination with the DigitalMicrograph™ 3.1 software (GATAN, Pleasanton, CA, USA). Images were adjusted for contrast and brightness using Adobe Photoshop CS (Adobe).

### Cell Sorting, RT-PCR, and Sequence Alignments

RT-PCR from isolated retinal ribbon synaptic cell types was performed using Rac3-EGFP and Lrrc55-EGFP transgenic mice expressing eGFP in cone photoreceptors and rod bipolar cells, respectively. For sorting of the respective eGFP positive cells, retinae were dissociated by papain digestion (20 U/ml; Worthington Biochemical, Lakewood, NJ, USA) at 37°C for 20 minutes with subsequent trituration and resuspension in FACS buffer (2% FCS, 2 mM EDTA in 0.1 M PBS, pH 7.4). Cells were sorted in a MoFlo High Speed Cell Sorter (Beckman Coulter, Krefeld, Germany) at the Nikolaus Fiebiger Center for Molecular Medicine, Erlangen, Germany, and collected in RLT buffer (Qiagen, Hilden, Germany) containing 1% β-Mercaptoethanol. Total RNA was isolated using the RNeasy Mini Kit (whole tissue) or the RNeasy Micro Kit (sorted cells) (Qiagen) and subjected to reverse transcription using random hexamers, M-MLV reverse transcriptase, 5x RT-buffer, a mixture of dNTPs, RNAsin (Promega, Mannheim, Germany) and 1 µg of total RNA (whole tissue) or complete RNA sample (sorted cells) in a total volume of 25 µl. For the polymerase chain reaction (PCR) 1 µl (whole tissue) or 2 µl (sorted cells) of cDNA was amplified in a final volume of 25 µl with 0.625 U of Taq DNA polymerase (Qiagen) and 10 pmol of each primer. Cycling conditions were: 45 cycles at 94°C for 45 seconds, 55°C for 45 seconds, and 72°C for 1.10 minutes followed by a final 72°C extension step for 10 minutes. Amplicon sizes were determined on 2% agarose gels stained with EtBr (Roth, Karlsruhe, Germany) and photographed using a computer assisted gel documentation system (DeVision G, Decon Science Tec, Hohengandern, Germany). Negative controls were treated as above without adding template. The identity of the PCR products was verified by DNA sequencing.

The following primers flanking intron 5/6 of the mouse Pclo gene (Pclo-201; ENSMUST00000030691) were used for RT-PCR and sequencing:

Forward primer: 5′-CTACCCTTCCTGAAGACCGT-3′; Reverse primer: 5′-GCTGTGGAATACTGCGGGGT-3′. Nucleotide and amino acid alignments from mouse, rat, cow, and human were generated with CLC Sequence Viewer 6 (CLC bio LLC, Cambridge, MA, USA).

### Electroretinography

The detailed procedure of measuring the ERG in mice has been described elsewhere [22]. Briefly, the animals were dark adapted overnight and all further handling was performed under deep red illumination. The mice were anesthetized by an intramuscular injection of 50 mg/kg ketamine (Ketavet®, Pfizer, Berlin, Germany) and 10 mg/kg xylazine (Rompun® 2%, Bayer, Leverkusen, Germany). A subcutaneous injection of saline solution (10 ml/kg, 0.9%) was administered to prevent desiccation. The pupils were dilated with a drop of tropicamide (Mydriaticum Stulln®, 5 mg/ml, Pharma Stulln, Stulln, Germany) and phenylephrine hydrochloride (Neosynephrin POS® 5%, Ursapharm, Saarbrücken, Germany). To measure the ERG, the ground gold needle electrode was placed subcutaneously at the basis of the tail, the reference gold needle electrodes were positioned subcutaneously next to the ears and the active contact lens electrodes (Mayo Corporation, Inazawa, Japan), internally covered with Corneregel® (Dr. Mann Pharma, Berlin, Germany), were placed on the cornea of each eye. To deliver the stimuli, a Ganzfeld Stimulator (Q450 SC, Roland Consult, Brandenburg, Germany) was used. Stimulation and data recording were controlled using the RetiPort system (Roland Consult). Initially, the dark adapted flash ERG was measured. The flash strength increased in eight steps (0.0002, 0.002, 0.0063, 0.02, 0.063, 0.2, 0.63, and 6.3 cd s/m^2^) and, depending on flash strength, 8 to 12 flashes were averaged. Flash duration varied between 5 µs and 5 ms depending upon the required total energy. After five minutes adaptation to 25 cd/m^2^ steady background light, photopic flash ERG measurements were performed. Flashes of five strengths (0.063, 0.2, 0.63, 2, and 6.3 cd s/m^2^) were superimposed on the background. At each flash strength, 20 responses were averaged. Off-line analyses of the responses were performed using custom-designed Matlab® (Mathworks, Ismaning, Germany) routines and Excel (Microsoft, Redmond, WA, USA) spreadsheets. From the scotopic flash ERG responses, the oscillatory potentials were extracted and discarded by using a variable filter procedure [22]. The amplitudes and latencies of the a- and b-waves were measured from the filtered responses. The a-wave amplitude was defined as the difference between the baseline level before stimulus onset and the minimum of the a-wave. The b-wave amplitude was defined as the difference between the a-wave minimum and the b-wave maximum. Latencies were defined as the time between stimulus onset and the minimum or maximum, respectively. The b-wave amplitude and latency of the photopic flash ERGs was measured in an analogous manner. Statistic differences between wt and Pclo-mutant mice were tested using ANOVA and Tukey’s post-hoc pairwise comparison tests. P-values <0.05 were considered significant.

## Results and Discussion

### Pclo Staining is Present at Retinal Ribbon Synapses in the Pclo-deficient Mouse

**Figure 1:**
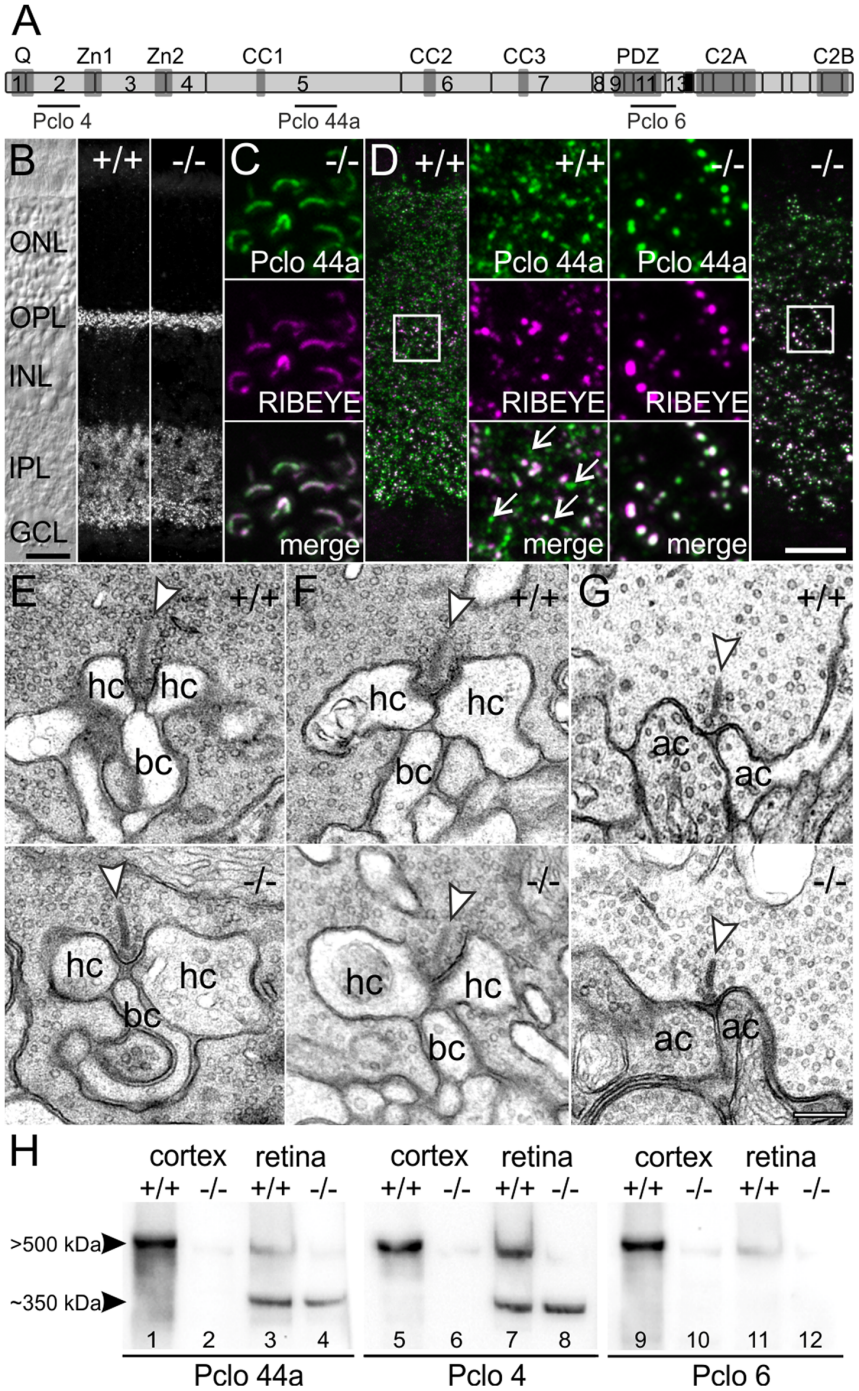
Presence of Pclo at retinal ribbon synapses in the Pclo-mutant mouse. **A**: Schematic representation of Pclo. The exons (numbered light gray boxes), interaction domains (dark gray boxes), and epitope locations for the three polyclonal antibodies Pclo 4, Pclo 44a, and Pclo 6 are shown. Exon 14 (black box) is deleted in the Pclo-mutant (−/−) mouse. **B**: Nomarski micrograph and images of vertical sections through wild-type (+/+) and −/− retina stained with Pclo 44a. **C**: Synaptic ribbons in the outer plexiform layer (OPL) of the −/− retina double labeled for Pclo (Pclo 44a; *green*) and RIBEYE (*magenta*). **D**: Inner plexiform layer (IPL) in the +/+ and −/− retina double labeled for Pclo (Pclo 44a; *green*) and RIBEYE (*magenta*). Arrows depict single Pclo positive puncta. **E-G**: Electron micrographs of rod (**E**) and cone (**F**) photoreceptor, and rod bipolar cell (**G**) ribbon synapses from +/+ and −/− retina. Arrowheads point to synaptic ribbons. **H**: Western blots of cortex and retina synaptosomal fractions from +/+ and −/− mice probed with the three different Pclo antibodies. ONL: outer nuclear layer; INL: inner nuclear layer; GCL: ganglion cell layer; hc: horizontal cell; bc: bipolar cell; ac: amacrine cell; kDa: kilo-Dalton. Scale bar in B: 20 µm, D: 10 µm, E–G: 200 nm.

In the rodent retina, Piccolo is present at conventional chemical synapses as well as ribbon-type synapses [16]. To study the retinal synaptic phenotype in mice lacking full-length Pclo (B6;129S6-Pclotm2Sud/J X Tg(CMV-cre)1Nagy), we first analyzed vertical sections through wt and Pclo-mutant retinae with an antibody against Pclo (Pclo 44a; Fig. 1*A,B*). Unexpectedly, in the Pclo-mutant retina, strong Pclo staining was observed in the two synaptic layers, i.e. the outer (OPL) and the inner (IPL) plexiform layer (Fig. 1*B*). In the OPL, the appearance of the photoreceptor ribbon synapses did not differ between wt and Pclo-mutant retina. In the IPL, the number of discrete Pclo puncta, representing individual synapses, was apparently reduced in the Pclo-mutant retina (Fig. 1*B*). This indicates that, while in the brain of the Pclo-mutant mouse most of Pclo is lost from synapses [*18*], in the retina only a subset of synapses, mainly in the IPL, is affected.

As the majority of chemical synapses in the OPL are photoreceptor ribbon synapses, Pclo seems to be present at these synapses in the Pclo-mutant retina. To confirm this, we double labeled photoreceptor ribbons with the Pclo44a antibody (Fig. 1*C*; green) and an antibody against RIBEYE (Fig. 1*C*; magenta) demonstrating a complete co-localization of the two proteins in the OPL of the Pclo-mutant retina (Fig. 1*C*; merge). In the IPL of wt retina, co-localized Pclo/RIBEYE puncta representing bipolar cell ribbon synapses, and single Pclo puncta representing conventional amacrine cell synapses [16], could be observed (Fig. 1*D*). In the IPL of Pclo-mutant retina, single Pclo puncta were missing, and only Pclo/RIBEYE puncta were detectable (Fig. 1*D*). Additional electron microscopical analysis of photoreceptor and bipolar cell ribbon synapses revealed no structural differences between wt and Pclo-mutant retinae (Fig. 1*E–G*). These data strongly suggest that photoreceptor and bipolar cell ribbon synapses express a Pclo variant which is not affected by the exon-14 deletion and therefore differs from the Pclo protein described for the brain [23]–[25].

In agreement with the previously published molecular weight for Pclo, the antibody Pclo 44a detected one prominent band of >500 kDa on Western blots of wt mouse cortex synaptosomal (P2) fractions (Fig. 1*H*; lane 1). In P2 fractions of wt retina, Pclo 44a labeling revealed an additional prominent band of ∼350 kDa, which is absent from cortex (Fig. 1*H* *;* lanes 1+3; see also [9]). In agreement with the reported loss of Pclo at conventional synapses [18], we could hardly detect the full-length Pclo band in P2 fractions of cortex and retina of the Pclo-mutant mouse (Fig. 1*H*; lanes 2+4). In contrast, the expression of the ∼350 kDa Pclo variant was comparable in wt and Pclo-mutant retinae (Fig. 1*H*; lanes 3+4), indicating the absence of the shorter Pclo variant at conventional synapses and its specific expression at retinal ribbon synapses. Since the expression of the shorter Pclo variant is apparently not affected by the deletion of exon 14, the longer (>500 kDa) and the shorter (∼350 kDa) Pclo variant most likely differ in their C-termini.

To confirm this, we performed Western blots of wt and Pclo-mutant cortical and retinal P2 fractions with antibodies directed against an N-terminal epitope (Pclo 4) and a C-terminal epitope (Pclo 6; Fig. 1*A,H*; lanes 5–12) of Pclo. Comparable to Pclo 44a labeling, Pclo 4 recognized the long Pclo variant in wt cortex and both the long and short Pclo variant in wt retina (Fig. 1*H*; lanes 5+7); in cortex and retina of the Pclo-mutant mouse, the long Pclo variant was barely detectable (Fig. 1*H*; lanes 6+8). The C-terminally binding Pclo 6 antibody detected only the long Pclo variant in wt cortex and retina, consistent with the lack of a large part of the C-terminus in the shorter, ribbon-specific Pclo variant (Fig. 1*H*; lanes 9–12).

### Alternative Splicing Generates a C-terminally Truncated Pclo Variant

**Figure 2:**
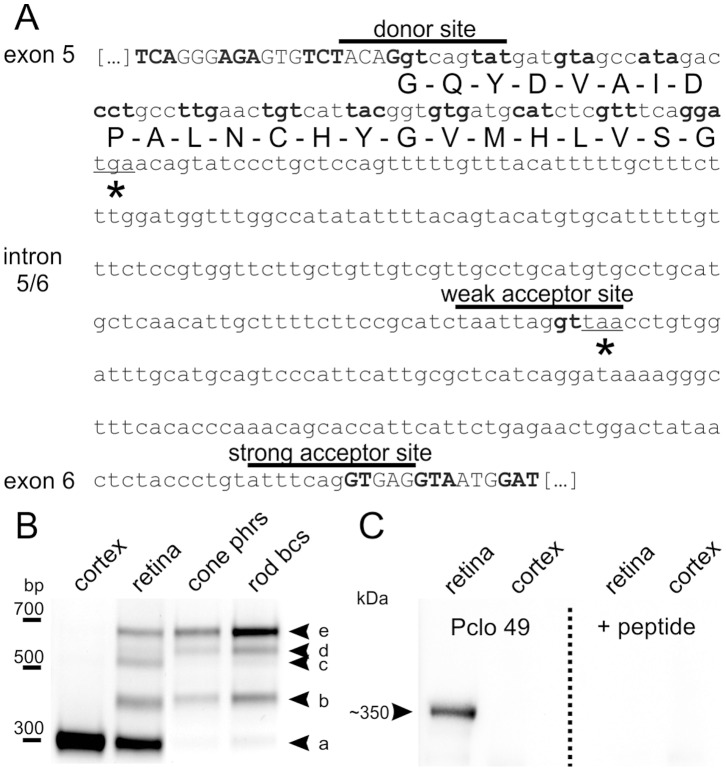
Intron retention generates a C-terminally truncated ribbon synapse specific Pclo variant. **A**: Nucleotide sequence of intron 5/6 in the *Pclo* gene (lower case letters) with flanking exon regions (capital letters). Codons are demarcated through alternating bold and non-bold letters, and the conventionally used donor and strong acceptor site, and a hypothetical alternative weak acceptor site are indicated with black lines. Utilization of the weak acceptor site as well as complete intron retention would result in in-frame stop codons (asterisks). The amino acid sequence used for the generation of Pclo 49 is displayed beneath the nucleotide sequence. **B**: RT-PCR of cDNA from cortex, retina, isolated cone photoreceptor (cone phrs) and rod bipolar cells (rod bcs) with primers flanking intron 5/6 in the *Pclo* gene. **C**: Western blot of wild-type retina and cortex synaptosomal fractions probed with Pclo 49 against the first 23 amino acids of intron 5/6 in the *Pclo* gene. Pclo 49 labels a ∼350 kDa band in the retina, but not in cortex (left panel). Pre-incubation of Pclo 49 with the antigenic peptide completely abolished the labeling (right panel). bp: base pairs; kDa: kilo-Dalton.

Next, we studied the cause for the Pclo truncation in retinal ribbon synapses. The epitope location of Pclo 6 predicts that the short Pclo variant lacks part of the C-terminus including the PDZ-domain (Fig. 1*A*). We therefore analyzed intronic regions upstream of exon 9 in the reported full-length Pclo transcript (Pclo-201; ENSMUST00000030691) with the web-based splice site analysis tool SplicePort [26] for hypothetical alternative splice sites, which may lead to premature stop codons. In addition to the conventionally used strong acceptor site, a possible weaker acceptor splice site was predicted to reside in intron 5/6 (Fig. 2*A*). Both the utilization of this alternative acceptor site as well as a complete retention of the 356 bp-long intron 5/6 would result in the presence of an in-frame stop codon leading to premature translation termination (Fig. 2*A*; asterisks). The calculated molecular weight of ∼330 kDa for this putative translation product matches the apparent MW of ∼350 kDa of the short retinal Pclo variant found in Western blots (Fig. 1*H*; lanes 3, 4, 7, 8).

To test whether alternative splicing in this region of Pclo actually occurs in the retina, we performed an RT-PCR analysis with exonic primers flanking intron 5/6 (expected bp: 319 without intron; 439 with predicted alternative splice site; 675 with retained intron). RT-PCR was performed with cDNA from total RNA and compared between cortex, whole retina, and isolated cone photoreceptor and rod bipolar cells (Fig. 2*B*). Amplification from cortical cDNA produced a single amplicon of ∼300 bp, confirming that the conventionally spliced transcript, which generates the >500 kDa Pclo variant (Fig. 2*B*; band a), constitutes the by far most abundant Pclo isoform. In retinal cDNA, however, we detected four additional amplicons of ∼400 bp, ∼550 bp, ∼600 bp, and ∼675 bp (Fig. 2*B*; bands b–e). Sequencing confirmed that band (b) corresponds to the predicted alternatively spliced Pclo transcript, and band (e) to a splice variant in which intron 5/6 is completely retained. Sequencing of bands (c) and (d) showed no relation to Pclo. Noteworthy is that both alternative transcript variants were preferentially expressed in retinal cell types containing ribbon synapses, i.e. cone photoreceptor and rod bipolar cells, whereas we detected only weak if any expression of the conventionally spliced Pclo variant in these cell types (Fig. 2*B*).

Verifying non-splicing of intron 5/6 at the transcript level with RT-PCR is problematic since amplicons containing the intron may also arise from possible contamination of the cDNA sample with genomic DNA. If, however, retention of intron 5/6 is indeed the mechanism which generates a truncated Pclo variant, the 5′-terminal part of the intron would be translated into protein. To verify the existence of a translation product derived from the alternative Pclo transcript at retinal ribbon synapses, we generated a polyclonal antibody (Pclo 49) against the first 23 amino acids encoded by intron 5/6 of the *Pclo* gene (Fig. 2*A*). On Western blots of wt retina and cortex P2 fractions, Pclo 49 recognized a high molecular weight protein band in retina but not in cortex (Fig. 2*C*). This protein band corresponds to the shorter, ribbon-specific Pclo variant detected with Pclo 44a and Pclo 4 (Figs. 1*H*; lanes 3, 4, 7, 8; 2*C*). Blocking Pclo 49 with the antigenic peptide used for immunization completely abolished the labeling on Western blots (Fig. 2*C*), demonstrating the specificity of the antibody Pclo 49. In summary, ribbon-specific alternative splicing of the Pclo transcript leads to a C-terminally truncated Pclo protein, which we named Piccolino. Coincidentally, the word Piccolino is not only an allusion to the smaller size of the truncated protein compared to the full-length variant, but also to Marco Piccolino, one of the first researchers describing the release of a depolarizing transmitter by photoreceptors in darkness [27].

### Piccolino is Present at Ribbon Synapses of the Retina and the Inner Ear

**Figure 3:**
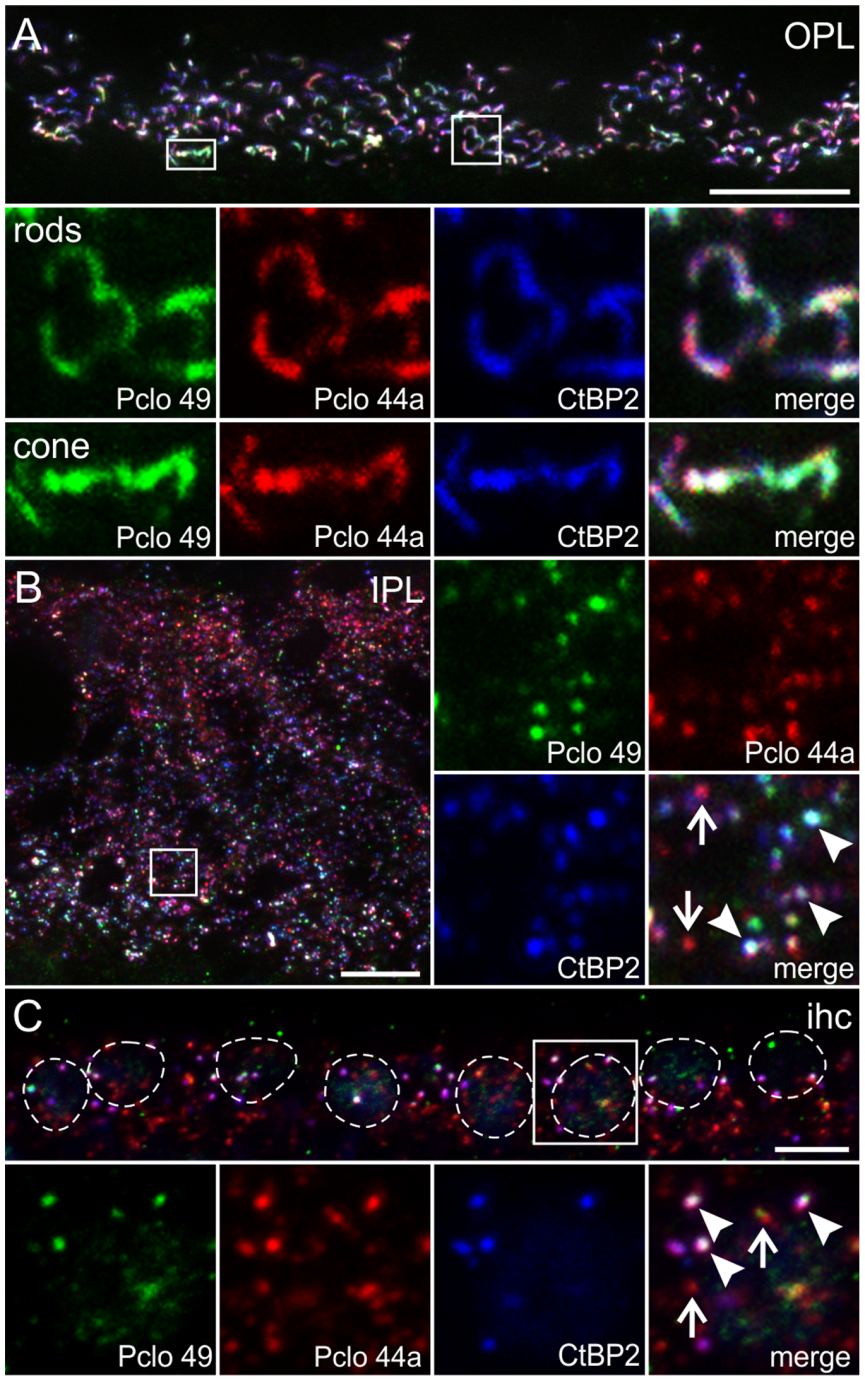
Localization of Piccolino at different types of ribbon synapses in the mouse. **A**: Outer plexiform layer (OPL) of +/+ retina triple labeled with Pclo 49 (labels Piccolino; *green*), Pclo 44a (labels Pclo and Piccolino; *red*), and an antibody against CtBP2/RIBEYE (*blue*). **B**: Inner plexiform layer (IPL) of +/+ retina triple labeled with Pclo 49 (labels Piccolino; *green*), Pclo 44a (labels Pclo and Piccolino; *red*), and an antibody against CtBP2/RIBEYE (*blue*). Arrowheads point to ribbon synapses, arrows demarcate Pclo 44a single stained conventional chemical synapses. **C**: Inner hair cells (ihc) triple labeled with Pclo 49 (labels Piccolino; *green*), Pclo 44a (labels Pclo and Piccolino; *red*), and an antibody against CtBP2/RIBEYE (*blue*). Nuclei (stained with DAPI, not shown) are circled with dotted lines. Arrowheads point to ribbon synapses, arrows demarcate conventional chemical synapses. Scale bar in A,B: 10 µm, C: 5 µm.

For a detailed analysis of Piccolino expression and localization in ribbon-type sensory synapses, we performed triple labeling experiments combining antibodies Pclo 49 (Fig. 3; green; stains only Piccolino), Pclo 44a (red; stains both Piccolino and Pclo), and an antibody against CtBP2/RIBEYE (blue; stains the ribbons) on vertical sections through wt mouse retina and on whole-mount preparations of the organ of Corti. In the retina, the three antibodies co-localized at ribbon synapses throughout the OPL, demonstrating the presence of Piccolino at rod and cone photoreceptor ribbon synapses (Fig. 3*A*). In the IPL, the high degree of co-localization between Piccolino (Pclo 49) and CtBP2/RIBEYE confirms the presence of Piccolino at bipolar cell ribbon synapses (Fig. 3*B*; arrowheads). Whereas single Pclo puncta (Pclo 44a) were present at amacrine cell synapses in the IPL (Fig. 3*B*; arrows), we did not detect single Piccolino (Pclo 49) or CtBP2/RIBEYE puncta in the IPL. In the organ of Corti, the three antibodies co-localized at ribbon synapses of inner hair cells (ihc; Fig. 3*C*; arrowheads). In addition, we found single Pclo puncta (Pclo 44a), most likely representing axodendritic efferent synapses (Fig. 3*C*; arrows; [28], [29]). Taken together, the results from the immunocytochemical experiments verify the presence of Piccolino across different sensory tissues – retina and organ of Corti – and across different types of ribbon synapses in four individual cell types – rod and cone photoreceptor cells, bipolar cells, and inner hair cells –, and indicate a specific role of Piccolino in ribbon synaptic function.

### Piccolino is the Prevalent Pclo Variant Expressed at Ribbon Synapses

**Figure 4:**
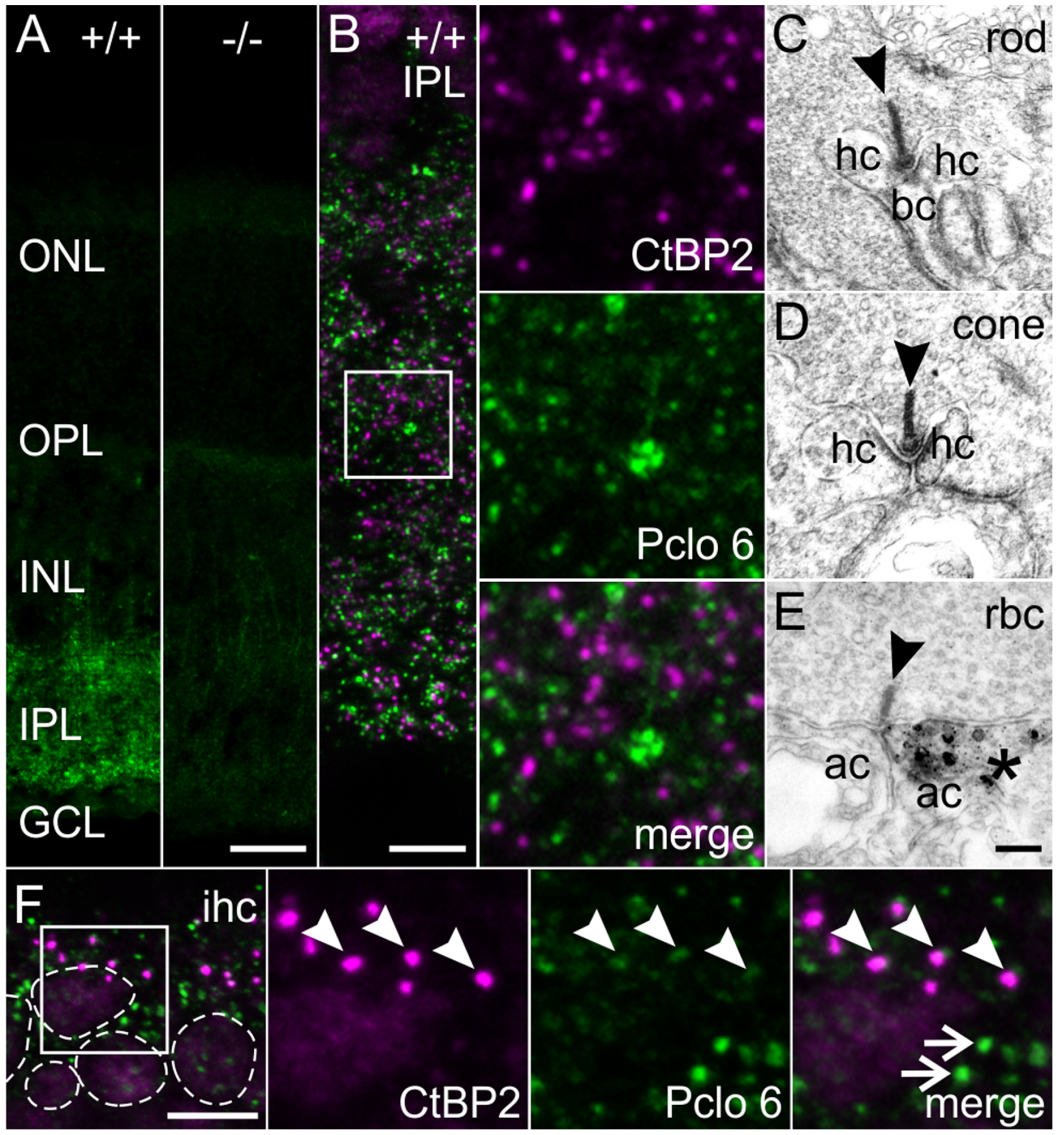
Localization of full-length Pclo at different types of ribbon synapses. **A**: Wild-type (+/+) and Pclo-mutant (−/−) retinae stained with the C-terminally binding Pclo 6 against full-length Pclo. **B**: Inner plexiform layer (IPL) of +/+ retina double labeled for full-length Pclo (Pclo 6; *green*) and CtBP2/RIBEYE (*magenta*). **C–E**: Pre-embedding immunoelectron micrographs of a rod photoreceptor (**C**), cone photoreceptor (**D**), and rod bipolar cell (rbc) ribbon synapse (**E**) in the +/+ retina stained with Pclo 6. Only amacrine cell synapses (**E**; asterisk) and never ribbon synapses (**C**–**E**; arrowheads) were stained for full-length Pclo. **F**: Inner hair cells (ihc) double labeled for full-length Pclo (Pclo 6; *green*) and CtBP2/RIBEYE (*magenta*). Nuclei (stained with DAPI, not shown) are circled with dotted lines. Arrowheads point to ribbon synapses, arrows demarcate conventional chemical synapses. ONL: outer nuclear layer; OPL: outer plexiform layer; INL: inner nuclear layer; GCL: ganglion cell layer. hc: horizontal cell; bc: bipolar cell; ac: amacrine cell. Scale bar in A,B: 20 µm, C-E: 200 nm, F: 5 µm.

Our RT-PCR analysis implied a virtual absence of the long Pclo variant from ribbon synapses (Fig. 2*B*). To show that Piccolino is not only ribbon-specific but also the predominant Pclo variant at ribbon synapses, we stained wt and Pclo-mutant retinae as well as whole-mount preparations of the organ of Corti with Pclo 6, the C-terminally binding Pclo antibody (Figs. 1*A*, 4). In the wt retina, Pclo 6 labeled synapses in the IPL but not in the OPL (Fig. 4*A*). This staining was absent in the Pclo-mutant retina (Fig. 4*A*), and additional double labeling experiments with Pclo 6 (green; Fig. 4*B*) and CtBP2/RIBEYE (magenta; Fig. 4*B*) confirmed the absence of the full-length Pclo variant at ribbon synapses in the IPL of wt retina. To exclude the possibility of epitope masking by chemical fixation, we repeated the staining on unfixed mouse retina and obtained the same result (data not shown). Finally, we confirmed the light microscopical findings with pre-embedding immunolabeling using the Pclo 6 antibody and electron microscopy, demonstrating the absence of full-length Pclo at photoreceptor and bipolar cell ribbon synapses (Fig. 4*C,D*), and its presence at amacrine cell synapses in the IPL (Fig. 4*E*). In the organ of Corti, Pclo 6 strongly labeled the presumed conventional axodendritic efferent synapses, as judged from the absence of CtBP2/RIBEYE labeling at these synapses (Fig. 4*F*; arrows). Sometimes we also detected weakly labeled Pclo 6 puncta in immediate vicinity of CtBP2/RIBEYE staining (Fig. 4*F*; arrowheads). These puncta might represent a tight spatial association of inner hair cell presynaptic ribbon sites with efferent synapses, although we cannot fully exclude the presence of the full-length Pclo at inner hair cell ribbon synapses. However, it is important to stress that staining for Piccolino at inner hair cell ribbon synapses was always much stronger than for full-length Pclo, indicating that Piccolino is also the predominant Pclo variant at inner hair cell ribbon synapses in the inner ear.

**Figure 5:**
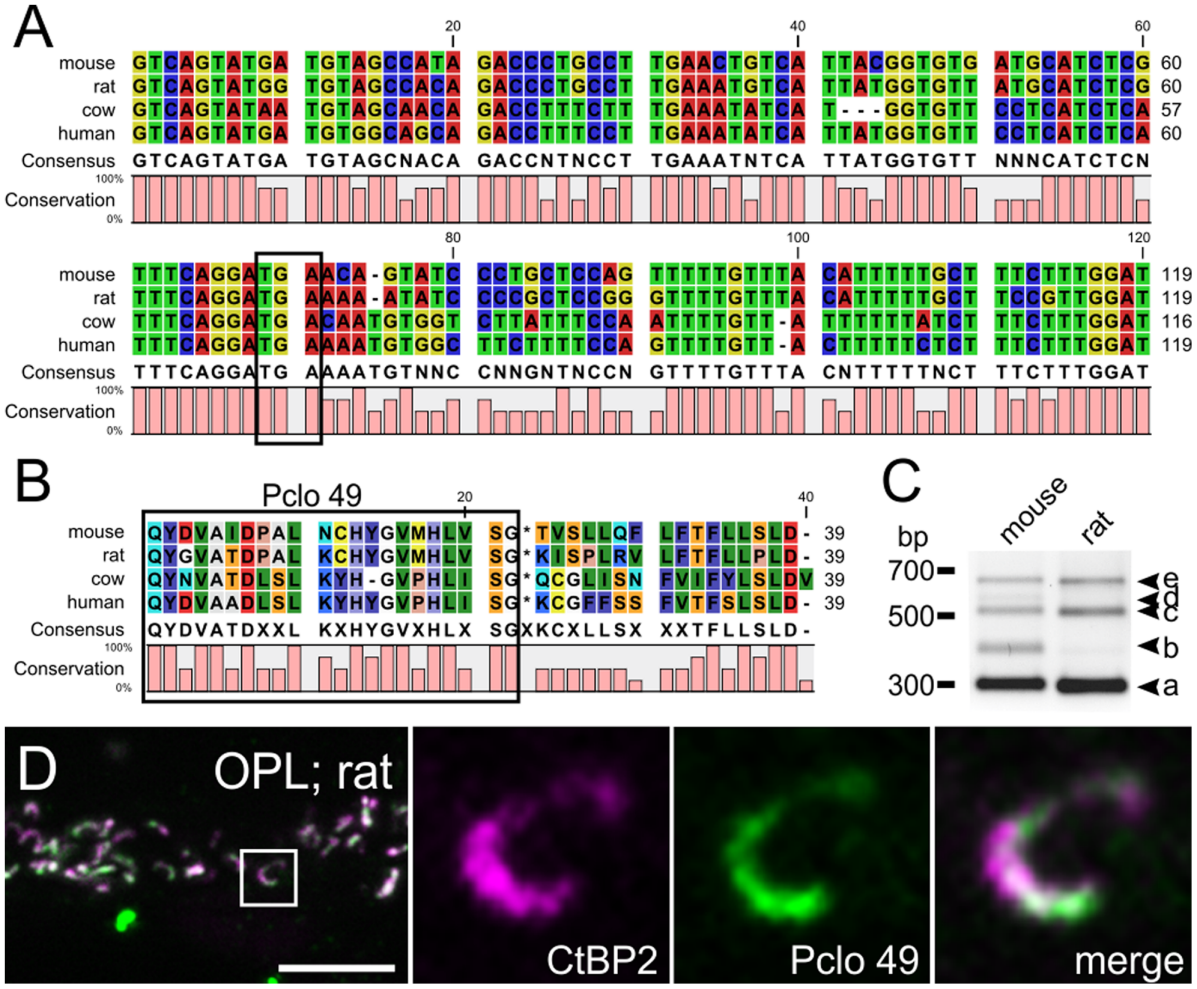
Piccolino and full-length Pclo expression in different species. **A**: Sequence comparison of the first 120 nucleotides of the Pclo intron 5/6 between mouse, rat, cow, and human. Note the 100% conservation of the stop codon in all four species (TGA; boxed region). **B**: Amino acid sequence comparison of the translation product derived from (A) between mouse, rat, cow, and human. The homology of the translated sequence (boxed region) ranges from 59% between mouse and cow, and 86% between mouse and rat. **C**: Comparative RT-PCR of mouse and rat retinal cDNA with primers flanking intron 5/6 of the *Pclo* gene (see also Figure 2). Like in the mouse retina, also in the rat retina four additional amplicons (b–e) were detected in addition to the strongly expressed conventionally spliced Pclo transcript (a), with (e) representing the completely retained intron 5/6 of the *Pclo* gene. **D**: Representative image of the outer plexiform layer (OPL) of PFA-fixed vertical sections through rat retina double stained with antibodies against CtBP2/RIBEYE (magenta) and Piccolino (Pclo 49; green). Scale bar in D: 5 µm.

At this point it has to be mentioned that Limbach et al. [10] reported a staining of rat photoreceptor ribbons with a C-terminally binding Pclo antibody, which is at odds with our findings that full-length Pclo seems to be absent from mouse photoreceptor ribbons. Species differences or methodological differences could be the reason for this discrepancy. Sequence alignments revealed a high conservation of the stop codon TGA in intron 5/6 of the *Pclo* gene between different species, i.e. mouse, rat, cow, and human, (Fig. 5*A*), suggesting the presence of Piccolino across different species. For the rat retina we could verify the existence of the alternative *Pclo* transcript with RT-PCR (Fig. 5*C*, *b+e*), and the new antibody Pclo 49 strongly stained photoreceptor ribbons in rat retinal cryostat sections (Fig. 5*D*). When we stained fixed and unfixed cryostat sections of rat and mouse retina with the C-terminally binding antibody Pclo 6, recognizing full-length Pclo, we found only occasionally weakly Pclo 6 positive ribbons in rat retina (data not shown) and no Pclo 6-labeled ribbons in mouse retina. Also in rat retina, the majority of ribbons were strongly labeled with the antibody Pclo 49, proving Piccolino expression at retinal ribbon synapses in different species (Fig. 5*D*).

Interestingly, amino acid sequence alignment of the resulting translation product of the retained intron 5/6 between different species shows high variation in the percentage of homology ranging from 86% (mouse and rat) to 59% (mouse and cow) (Fig. 5*B*). This implies that the short C-terminal sequence of Piccolino which differs from the long Pclo variant may not exert any physiological function other than truncation of Pclo at this position.

### Basal Transmission at Photoreceptor Ribbon Synapses is Unaffected by the Deficiency of Full-length Pclo

**Figure 6:**
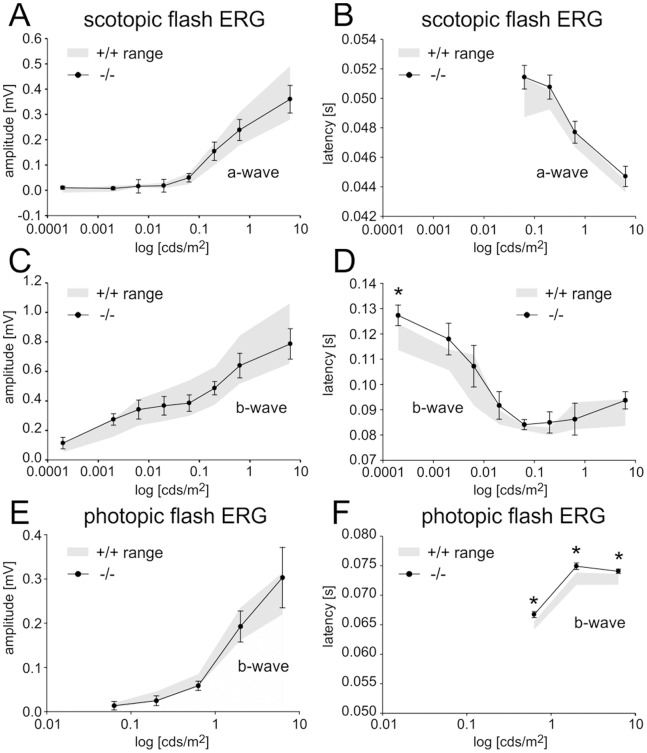
Scotopic and photopic ERG recordings from wild-type (+/+) and Pclo-mutant (−/−) mice. **A**: The mean (± sd) amplitude of the scotopic a-wave of +/+ (*gray*) and −/− mice (filled circles) increased with increasing flash intensity. There was no difference between +/+ and −/− mice. **B**: The mean (± sd) latency of the scotopic a-wave of +/+ and −/− mice decreased with increasing flash intensity. There was no significant difference between +/+ and −/− mice. **C**: The mean (± sd) amplitude of the scotopic b-wave of +/+ and −/− mice increased with increasing flash intensity in both +/+ and −/− mice. **D**: The mean latency of the scotopic b-wave decreased with increasing flash intensity in both +/+ and −/− mice. The asterisk indicates a significant difference between +/+ and −/− mice at a flash strength of 0.0002 cd.s/m^2^ (p<0.05). **E**: The mean (± sd) amplitude of the photopic b-wave increased with increasing flash intensity. There was no difference between +/+ and −/− mice. **F**: The mean latency of the photopic b-wave increased with increasing flash intensity. The b-wave latency of −/− mice was significantly increased (p<0.0001) by approximately 2 ms.

If Piccolino is the predominant ribbon synaptic Pclo variant, deficiency of full-length Pclo should not affect photoreceptor ribbon synaptic transmission. However, post-receptoral function may be altered because of changes in the conventional amacrine cell synapses in the IPL. To test this hypothesis, we performed electroretinographic (ERG) recordings from wt and Pclo-mutant mice (Fig. 6). The a-wave in the ERG predominantly reflects the photoreceptor ionic currents, and the b-wave primarily reflects the ON bipolar cell activity, which is a good readout for photoreceptor ribbon synaptic transmission and function. We found that both the amplitudes (Fig. 6A) and latencies (Fig. 6B) of the scotopic (mainly rod driven) a-wave were very similar in wt and Pclo-mutant mice, demonstrating that phototransduction is not disturbed in the Pclo mutant. Under scotopic conditions, the amplitudes of the b-wave were also comparable between wt and Pclo-mutant mice (Fig. 6C). The latency of the b-wave in the Pclo-mutant mice was slightly but significantly prolonged at a flash intensity of 0.0002 cd.s/m^2^ (p<0.05); at all other flash intensities, the b-wave latency was comparable between wt and Pclo-mutant mice (Fig. 6D). Consistent with the scotopic data, the amplitudes of the photopic b-waves did not differ in the two genotypes (Fig. 6E). The photopic (cone driven) b-wave was slightly but significantly (p<0.001) delayed by about 2 ms in the Pclo-mutant mice at all flash intensities (Fig. 6F). We propose that this delay is caused by the influence of Pclo-deficient amacrine cell synapses on the activity of bipolar cells, being in line with the contribution of third order neurons, like amacrine cells, on the ERG b-wave [29]–[32].

Applying the ERG as readout for retinal function, we cannot completely rule out that the lack of full-length Pclo has subtle functional effects on photoreceptor synaptic transmission which might stay undetected with the ERG. Nonetheless, comparing the functional synaptic phenotype of the Pclo-mutant (this study) and the Bsn-mutant mice [6], we interpret the unaltered ERG recordings in the Pclo-mutant mice as physiological support for a minor role or even complete absence of full-length Pclo at photoreceptor ribbon synapses, as indicated by our molecular analyses.

### Putative Lack of Interaction Sites for CAZ Proteins like Bsn and Munc13 in the C-terminally Truncated Piccolino

**Figure 7:**
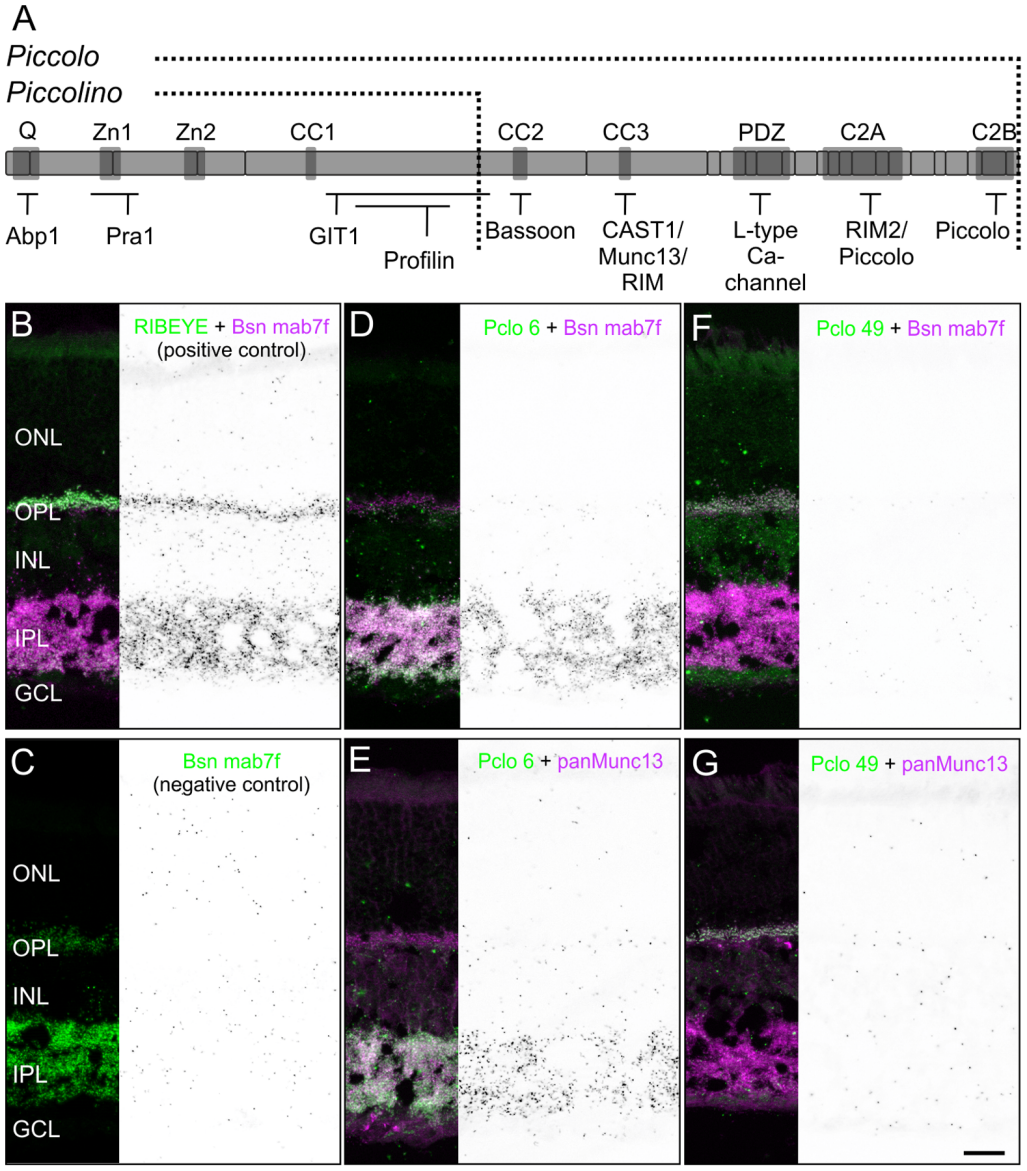
Missing interactions of Piccolino with Bsn and Munc13. **A**: Schematic representation of full-length Pclo with its interaction domains (*dark gray* boxes) and known binding partners. The C-terminally truncated Piccolino lacks the C-terminal interactions. **B–G**: *In situ* proximity ligation assays (PLA) on vertical sections through wild-type retina (black and white panels) with corresponding fluorescence stainings. Positive control: interaction of RIBEYE and Bsn with the antibodies RIBEYE (green) and Bsn mab7f (magenta; **B**). Negative control: antibody Bsn mab7f (green) alone (**C**). Interaction of full-length Pclo with Bsn (**D**) and Munc13 (**E**) probed with the antibodies Pclo 6 (green), Bsn mab7f (magenta), and panMunc13 (magenta). Interaction of Piccolino with Bsn (**F**) and Munc13 (**G**) probed with the antibodies Pclo 49 (green), Bsn mab7f (magenta), and panMunc13 (magenta). ONL: outer nuclear layer; OPL: outer plexiform layer; INL: inner nuclear layer; IPL: inner plexiform layer; GCL: ganglion cell layer. Scale bar: 20 µm.

Several interacting partners of Pclo have been identified in various neuronal and non-neuronal tissues, including Bsn [17], RIMs [17], [33], Munc13 [17], ELKS/CAST [34], and an L-type Ca^2^^+^ channel [35], suggesting the involvement of Pclo in the coordination of exo- and/or endocytosis at chemical synapses. The binding domains for these CAZ proteins all reside in the C-terminal portion of the full-length Pclo variant (Fig. 7*A*). As this part is missing in Piccolino, it can be assumed that these interactions do not take place at ribbon-type synapses. To support this, we chose to perform *in situ* proximity ligation assays (PLA; [36]) on vertical sections through wt mouse retina. In PLAs, oligonucleotide-tagged secondary antibodies are linked with circle-forming oligonucleotides when two antigens, detected by two primary antibodies derived from different species, are in close proximity (<40 nm) to each other. After ligation of the two linker oligonucleotides, rolling circle amplification with simultaneous hybridization of complementary fluorophore-tagged oligonucleotide probes results in fluorescent puncta at the site of interaction. Thus, an absence of PLA signal for Piccolino with arciform density proteins in the OPL, despite their close spatial proximity at the photoreceptor ribbon complex [9], would be a strong indicator for a non-existing interaction. The applicability of PLAs on retinal slices was demonstrated by Venkatesan et al. [37] for the interaction of RIBEYE with GCAP2. Because monoclonal mouse antibodies against ELKS/CAST, RIM2, and the L-type Ca^2^^+^ channel were not available, PLAs for full-length Pclo and Piccolino in combination with these proteins were technically not feasible.

As positive control we first tested the known interaction of RIBEYE and Bsn [9]. Both proteins are colocalized at ribbon synapses in the OPL and IPL despite the predominating RIBEYE-labeling in the OPL and the predominating Bsn-labeling in the IPL, which is due to the antibody combination used in this experiment (RIBEYE and Bsn mab7f; Fig. 7*B*). Still, this antibody combination produced a strong PLA signal in the two synaptic layers of the retina, representing interaction of the two proteins at photoreceptor and bipolar cell ribbon synapses (Fig. 7*B*). Omitting either one of the antibodies resulted in the almost complete absence of any signal, proving the specificity of the PLA (Fig. 7*C*). A combination of Pclo 6, recognizing the full-length Pclo variant, and antibodies against Bsn or Munc13 produced strong signals in the IPL, but not the OPL (Fig. 7*D,E*), indicating an expected interaction of these proteins at conventional amacrine cell synapses. The latter findings are well in agreement with published data on full-length Pclo interactions with CAZ proteins [*17*], and the missing PLA signal in the OPL corroborates the virtual absence of full-length Pclo from retinal ribbon synapses.

As predicted from the lack of CAZ binding domains in Piccolino, testing the interaction of Piccolino (Pclo 49) with Bsn or Munc13 resulted in only very few and evenly distributed PLA puncta across the retina, but not in any specific signal in the synaptic layers (Fig. 7*E,F*). This indicates that Piccolino does not interact with these CAZ proteins, further implying that interactions with the L-type Ca^2^^+^ channel, RIM2, and ELKS/CAST may not exist either (Fig. 7*A*).

Due to the putative lack of interactions, we assume that Piccolino is unlikely to play a significant role in synaptic vesicle exocytosis at ribbon synapses. Instead we propose that an evolutionary switch from the expression of the full-length Pclo to the expression of a Pclo variant lacking the above mentioned interactions, might have facilitated the physical three-dimensional extension of the active zone into the cytoplasm in ribbon synapse containing sensory neurons. Moreover, in the N-terminal portion of Pclo, which is shared by Piccolino, reside the binding domains for Abp1 [38], Pra1 [25], GIT1 [39], and Profilin [24], proteins which are suggested to regulate actin dynamics, membrane trafficking, and synaptic vesicle endocytosis at AZs (Fig. 7*A*). Photoreceptor ribbon synapses promote ongoing cycles of exo- and endocytosis to faithfully transmit a wide range of stimulus intensities, and the efficient resupply and replenishment of synaptic vesicles for release is an essential function of the ribbon. Thus, it is tempting to speculate that Piccolino plays a role in these processes. With the identification of Piccolino, a novel Pclo splice variant specifically expressed at retinal ribbon synapses, the stage is set for further functional studies of the ribbon in general and of Piccolino in particular.
